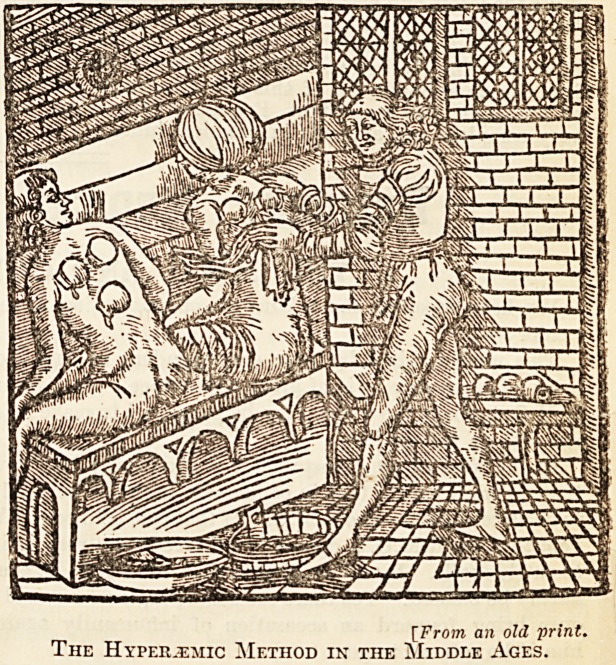# The Hyperæmic Method of Treatment.—I

**Published:** 1909-01-02

**Authors:** 


### ORTHOPÆDICS.

### THE HYPERÆMIC METHOD OF TREATMENT.—I.

Of late years considerable interest has been aroused in what is called the "Bier method" of treatment, that is to say treatment by means of hyperæmia. To orthopædists this method of treatment is a very valuable auxiliary, and it is one that should be better known to the general practitioner than at present it appears to be. It is not a new method. The old cupping-glass and the constriction mentioned by Hippocrates, Avicenna, and Rhazes were after all forerunners of the modern suction cup and Bier's bandage, but while admitting the priority right that undoubtedly belongs to the practitioners who first made use of the old methods, it is only just to acknowledge the debt that the profession owes to Professor Bier for having popularised the hyperæmic method and put it upon a rational footing. In Germany it has now been used for many years. Bier himself tried it fully while chief of the surgical clinic at Greifswald, and later on at Bonn, and at present the Mecca of the treatment is the Royal Surgical Clinic at Berlin, where, under the Professor's own immediate supervision a special "hyperæmic department" annually deals with many hundreds of patients. In America the method has been extensively tried, and has won deserved praise. With us in England it is by no means so well known as it ought to be, and its possibilities are not sufficiently well recognised. The result is that, partly owing to want of accurate knowledge of the technique, and partly owing to want of discrimination in the selection of cases, the recorded results with us are not so good as elsewhere. There is, therefore, a tendency to belittle this method of treatment, and to adopt an attitude of mild scepticism with regard to Continental and American reports. This is exceedingly unfortunate. The method is not properly taught to students, and is rarely used except in hospital practice, and in many quarters there is still an impression that it is complicated, demands expensive and cumbersome apparatus, and is only useful in exceptional cases.

### The Limitations of the Method.

This is far from being the case. It is, of course, not to be wondered at that a new method of treatment should have its hyper-enthusiastic supporters who by their exaggerated claims attempt to convince everyone of its supreme usefulness and its superiority over any and every other method in existence. The Bier method has not escaped these fanatical supporters. It has been hailed as a panacea for every imaginable disorder, from a sick headache to a generalised peritonitis. Examined critically and calmly it is soon apparent that many of these extravagant claims cannot be supported. But, at the same time, it is clear that the method has a very wide range of usefulness both in chronic and acute conditions. So far as orthopædic surgery is concerned it will be found a method eminently worthy of trial in inflammatory conditions of the joints and tendon sheaths, in tubercular lesions, septic conditions, and in many other cases. The practitioner will, of course, have to discriminate between cases that are likely to be benefited by the treatment and those in which the application of the method will do no good and is likely to do harm. At first this is not easy, but after seeing the effects of treatment in a few cases, and carefully noting the results, it is possible to tell more or less accurately what is the class of lesion in which the treatment may be expected to prove beneficial. Comparatively little practice is necessary to master the technique of the method, which is exceedingly simple and may be summarised in a few lines.

### The Apparatus Required.

The instruments which are necessary for carrying out the treatment are exceedingly simple, and by no means expensive.

[From an old print.

The Hyperæmic Method in the Middle Ages.

The induction of hyperæmia by means of hot-air baths and vapour cabinets is, of course, not a method suitable for use in private practice. It can only be successfully carried out in properly fitted up out-patient departments, where skilled assistance is at hand. It is, however, not the best method of inducing hyperæmia, and in private practice the simpler suction or constriction methods will be found much more useful and practically much more successful.

Suction hyperæmia is produced by the method of which the ordinary dry cupping is a familiar example. By decreasing the amount of air in a closed cup applied over the inflamed part, the tissues are sucked into the hollow of the cup, and the part is rendered hyperæmic owing to the increased blood flow towards it. This local hyperæmia acts in two ways chiefly. It produces an increase of tissue metabolism at the affected site, and a largely increased exudation of leucocytes into the inflamed tissues. In other words it produces an exaggeration of the natural defensive mechanism against local infection—dilatation of vessels, exudation, reaction, and hyperæmia. By constriction a similar condition is produced by damming back the venous return, as it were with a bandage encircling the limb some distance above the site of the lesion. It is unnecessary here to enter into the interesting questions of pathology raised by both methods,* but it may be pointed out that to a large extent cure by hyperæmia is Nature's cure, and what the surgeon does when he uses the Bier method is merely to help Nature towards concentrating her defensive forces upon the attacked spot.

For the suction method (which on the whole is less applicable to joint lesions) special suction cups, with an exhaust ball and rubber tube attached, are required. These cups are made of strong, hard, annealed white glass. They can be sterilised by immersion in antiseptic fluids, or, better still, by boiling. The rubber parts must be disinfected after use by immersion in antiseptic solutions, as they will not stand boiling. These cups are made in various shapes and sizes, and can be obtained from any German surgical instrument maker. The writer has lately used several excellent cups supplied by Messrs. Down Bros., of St. Thomas's Street, Borough, from which firm all the Bier apparatus may be obtained. It is desirable to keep at least two sizes of cups handy, a large and a small one, and also a strong suction cup fitted with a special exhaust syringe. The cups range in price from half-a-crown upwards, according to size.

For the constriction method all that is required is an elastic bandage. Special Bier's bandages, as used at Professor Bier's clinic, may be obtained. These are made of thin rubber, plain or cloth covered, and are about two and a quarter inches broad and three feet long. A piece of broad garter elastic serves admirably in most cases, and a length of Martin's bandage does equally well. Half a dozen of such bandages, whichever variety is selected, should be provided, and one used for each patient, as it is difficult and indeed unnecessary to sterilise them. An ordinary cloth bandage should never be employed for inducing constriction hyperæmia.

* Professor Bier's text-book on Hyperæmia (edition 1908) gives the latest and best information on the modifications of the method, and besides much useful information regarding the selection of cases and the general application of the method. The English translation (American edition, 1906) is really an abbreviation of this work under the editorship of Professor Bier's assistant, Professor Schmieden, but is very complete and abounds in practical hints.

## Figures and Tables

**Figure f1:**